# Supplementary material for: Validated ligand geometries for macromolecular refinement restraints and molecular mechanics force fields
Source: bioRxiv. 2025 Aug 2:2025.08.01.668229. Preprint. [Version 1] doi: 10.1101/2025.08.01.668229 (PMC12324484; doi:10.1101/2025.08.01.668229)
Supplement: 1 [file NIHPP2025.08.01.668229V1-supplement-1.pdf]

bioRxiv preprint doi: https://doi.org/10.1101/2025.08.01.668229; this version posted August 2, 2025. The copyright holder for this preprint (which was not certified by peer review) is the author/funder. This article is a US Government work. It is not subject to copyright under 17 USC 105 and is also made available for use under a CC0 license.

Supplementary

Table S1:

Size	PERFECT	PERFECT (side chain)	GRAND	GRAND (side chain)	OK	OK (reasonable std)	FAIL	Total success	Total Calculated
10	699	12	738	67	136	115	630	1767	2397
20	3725	36	3387	478	1353	1113	3899	10092	13991
30	3797	8	3388	134	2422	2129	5626	11878	17504
40	2033	6	1887	57	2032	1808	3273	7823	11096
50	556	0	481	42	733	620	1190	2432	3622
60	158	2	120	13	202	201	532	696	1228
70	58	1	28	1	55	98	258	241	499
80	13	0	7	1	28	23	78	72	150
90	5	0	8	1	14	12	31	40	71
100	1	0	3	0	6	7	20	17	37
110	0	0	1	0	2	1	9	4	13
120	0	0	0	0	1	3	6	4	10

Table S2:

Charge	PERFECT	PERFECT (side chain)	GRAND	GRAND (side chain)	OK	OK (reasonable std)	FAIL
-3	7.40%	0.15%	6.75%	2.60%	11.64%	6.25%	65.22%
-2	16.85%	0.15%	13.92%	2.59%	14.38%	11.05%	41.07%
-1	28.08%	0.42%	26.56%	6.96%	14.40%	12.29%	11.30%
0	21.79%	0.06%	19.67%	0.31%	13.85%	12.55%	31.78%
1	19.98%	0.37%	20.54%	1.02%	9.16%	10.18%	38.76%

Table S3:

bioRxiv preprint doi: https://doi.org/10.1101/2025.08.01.668229; this version posted August 2, 2025. The copyright holder for this preprint (which was not certified by peer review) is the author/funder. This article is a US Government work. It is not subject to copyright under 17 USC 105 and is also made available for use under a CC0 license.

Method	Mogul answer		
**PM6-D3H4**	GRAND		1352
**PM6-D3H4**	GRAND	side chain	166
**PM6-D3H4**	OK		8501
**PM6-D3H4**	OK	reasonable std	10347
**PM6-D3H4**	OK	side chain	346
**PM6-D3H4**	PERFECT		4346
**PM6-D3H4**	PERFECT	side chain	28
		TOTAL	25086

Original GeoStd Contents
ATOMN 5 [PSU]
ATOMP 456
ATOMS 9 [BMA, FUC, NGA, NDG, NAG, XLS, GAL, SIA, MAN]
HETAC 2 [NCO, IRI]
HETAI 4
HETAIN 55
HETAS 1
Total 532
